# Osseous Union of Deciduous Teeth

**Published:** 1842-12

**Authors:** 


*Osseous Union of Deciduous Teeth.*—A right lateral incisor and cuspidatus of the lower jaw, the crowns of which, are firmly united, were presented to us some months since, by Mr. Samuel Townsend, dentist, of Baltimore. The first has no root, it having been destroyed by the operations of the economy, to give place to the tooth that was to succeed it, and but for its osseous connection with the cuspidatus, it would very soon have dropped out; the second has a root, but the whole length of the side of it next the lateral incisor, is rough and partially destroyed. These teeth were extracted by Mr. Townsend to make room for a permanent lateral incisor that had come up behind the dental circle.

This is the first instance of osseous union of deciduous teeth which we recollect to have ever seen, and its occurrence between a lateral incisor and cuspidatus—the time for the dentition of the first being from seven to twelve months earlier than for that of the latter, renders it very remarkable. The eruption of both must have been effected at the same time.

